# Dual (pH- and ROS-) Responsive Antibacterial MXene-Based Nanocarrier for Drug Delivery

**DOI:** 10.3390/ijms232314925

**Published:** 2022-11-29

**Authors:** Wei-Jin Zhang, Shuwei Li, Yong-Zhu Yan, Sung Soo Park, Anandhu Mohan, Ildoo Chung, Suk-kyun Ahn, Jung Rae Kim, Chang-Sik Ha

**Affiliations:** 1 Department of Polymer Science and Engineering, School of Chemical Engineering, Pusan National University, Busan 46241, Republic of Korea; 2 School of Chemical Engineering, Pusan National University, Busan 46241, Republic of Korea; 3 Division of Advanced Materials Engineering, Dong-Eui University, Busan 47340, Republic of Korea; 4 Department of Industrial and Environmental Engineering, Gachon University, Seongnam 13120, Republic of Korea

**Keywords:** MXene, ROS-responsive, pH-responsive, antibacterial activity, doxorubicin, drug delivery

## Abstract

In this study, a novel MXene (Ti_3_C_2_T_x_)-based nanocarrier was developed for drug delivery. MXene nanosheets were functionalized with 3, 3′-diselanediyldipropionic acid (DSeDPA), followed by grafting doxorubicin (DOX) as a model drug to the surface of functionalized MXene nanosheets (MXene-Se-DOX). The nanosheets were characterized using scanning electron microscopy, atomic force microscopy (AFM), transmission electron microscopy, energy-dispersive X-ray spectroscopy (EDX), nuclear magnetic resonance spectroscopy, Fourier transform infrared spectroscopy, X-ray photoelectron spectroscopy, X-ray diffraction, and zeta potential techniques. The drug-loading capacity (17.95%) and encapsulation efficiency (41.66%) were determined using ultraviolet–visible spectroscopy. The lateral size and thickness of the MXene nanosheets measured using AFM were 200 nm and 1.5 nm, respectively. The drug release behavior of the MXene-Se-DOX nanosheets was evaluated under different medium conditions, and the nanosheets demonstrated outstanding dual (reactive oxygen species (ROS)- and pH-) responsive properties. Furthermore, the MXene-Se-DOX nanosheets exhibited excellent antibacterial activity against both Gram-negative *E. coli* and Gram-positive *B. subtilis*.

## 1. Introduction 

MXene (Ti_3_C_2_Tx) is a new category of materials and has attracted increasing attention from scientists since it was proposed by Gogotsi et al. [1,2]. It can be obtained by selectively etching the A-layer of MAX-phase ceramics, where M denotes a transition metal, A denotes a IIIA or IVA group element, X denotes carbon or nitrogen, and T denotes an -O, -OH, and/or-F group on the surface of MXene [3,4]. In recent years, two-dimensional (2D) MXenes have attracted significant research interest owing to their ultrathin planar nanostructure and intriguing physiochemical performance. Initially, the application of MXene primarily focused on electromagnetic interference (EMI) shielding, energy storage, and batteries [5,6]. Recently, the use of MXene for biomedical applications has been reported in the fields of photoacoustic (PA) imaging [7], anticancer treatment, and antibacterial activity [8]; however, cell viability, vector endocytosis ability, controllable release of a drug, tumor eradication capacity in vivo, and other biological applications such as biosensors should be investigated at the cellular and animal levels. 

Although many researchers have studied tumor treatments, malignancy remains one of the main threats to human health [9]. Well-known conventional cancer treatment modalities fall into three main categories, namely, surgery, radiotherapy, and drug therapy [10,11]. Notably, drug treatment has undergone three revolutions: chemotherapy (chemotherapeutic drugs comprising 5-fluorouracil (5-FU), paclitaxel (PTX), camptothecin (CPT), and doxorubicin (DOX)), targeted therapy, and immunotherapy modalities [12].

The major obstacles to the successful implementation of theranostics include unexpected release [13,14], the degradation of devices, burst release [15], and the lack of tumor homing [16] of some nanodevices. Chemotherapeutic drugs are well known for their high toxicity and poor water solubility [17]. Therefore, the premature release of chemotherapy drugs poses a serious risk to patients, decreases treatment effectiveness, causes irreversible damage to normal tissues, and significantly increases the burden on different aspects [18], such as the body’s metabolism and family finances. To address these issues, a drug delivery system (DDS) with precise release and good tumor homing that can prevent the unexpected release of a drug during blood circulation and increase the utilization rate of chemotherapeutic drugs has become a top priority. In particular, DDSs with stimuli-responsive properties under endogenous and exogenous environmental stimuli are urgently needed [16,19].

In normal tissues, the endothelial cells of capillaries are densely stacked with structural integrity, and it is difficult for invasive substances to pass through the walls of the vessel [20]. Nevertheless, tumor tissues have numerous capillaries with structural incompleteness, a broad gap in the vascular wall, and no lymphatic reflux, which result in the effortless permeation of invasive substances such as nanocarriers into cancer cells, simultaneously causing drug enhanced permeability and retention (EPR) effects at the tumor site. Furthermore, the premature release of chemotherapeutic drug molecules can be easily detected by antibodies and captured by macrophages during blood circulation [21]. In this regard, the covalent bonding of anticancer drugs to MXene nanodevices has been shown to effectively reduce adverse effects [22], prevent immune system destruction, and rapidly penetrate tumor cell membranes [23,24,25]. 

**Scheme 1:**
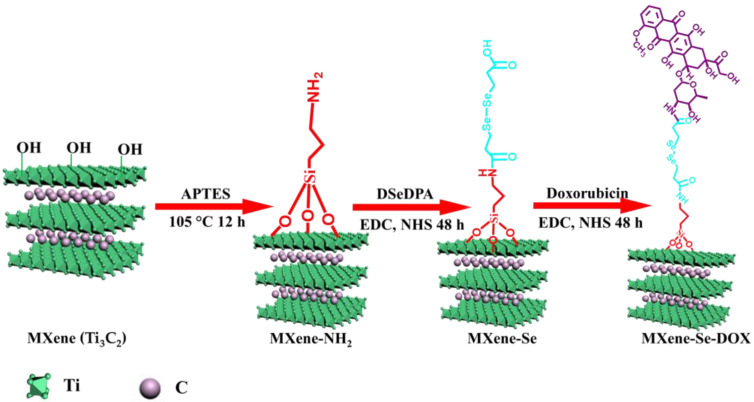
Schematic illustration of the synthesis process of MXene-Se-DOX nanosheets.

Therefore, in this study, we focused on the biomedical applications of MXene, including the specific delivery of a chemotherapeutic drug, and explored its antibacterial properties. To achieve this, we used MXene-based nanocarriers (MXene-Se-DOX) prepared by conjugating MXene with the chemotherapeutic drug DOX using 3,3’-diselenodiyldipropionic acid (DSeDPA) as a crosslinking agent (Scheme 1). DOX grafting on the surface of MXene via a covalent bond has many advantages compared with the physical encapsulation of a drug, including a reduction in the risk of the unexpected release of DOX and an improvement in the release controllability of a drug by external environmental stimuli. The MXene-Se-DOX nanosheets showed remarkable ROS-responsive behavior and pH-responsive behavior in vitro, as well as superior antibacterial activity at a constant concentration of 6.085 mg/mL. MXene-based nanocarriers are promising candidates for DDS.

The antibacterial MXene-based nanocarrier for drug delivery was prepared in three steps as follows: Initially, 3-aminopropyltriethoxysilane (APTES) was conjugated to the surface of MXene to introduce the amino group (MXene-NH_2_). Afterward, the carboxyl group of DSeDPA was reacted with the APTES-functionalized MXene to endow the carrier with ROS-responsive properties (MXene-Se). Finally, DOX was grafted onto the surface of MXene-Se nanosheets by condensation polymerization in the presence of 1-(3-dimethylaminopropyl)-3-ethylcarbodiimide hydrochloride (EDC) and 1-hydroxy-2,5-pyrrolidinedione (NHS) crosslinking agents.

## 2. Results and Discussion

### 2.1. FTIR and Raman Spectra

**Figure 1:**
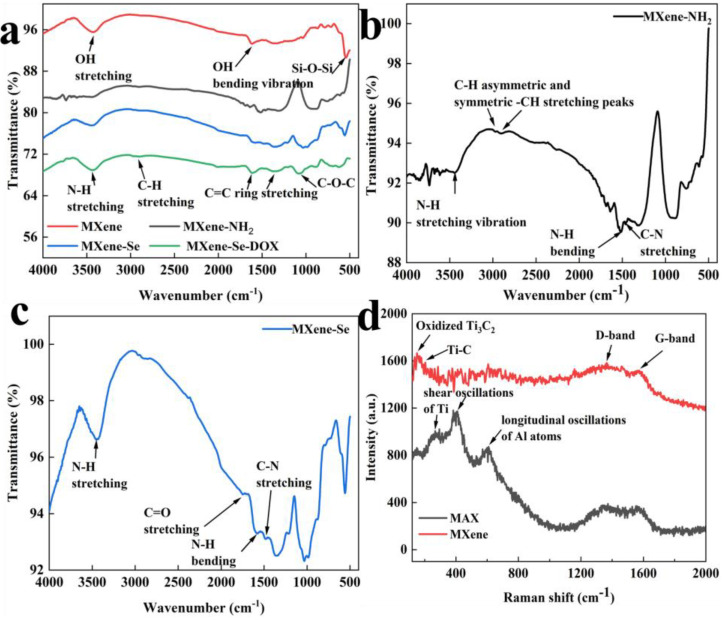
(**a**) FTIR spectra of MXene, MXene-NH_2_, MXene-Se, and MXene-Se-DOX. (**b**) Magnified FTIR spectrum of MXene-NH_2_. (**c**) Magnified FTIR spectrum of MXene-Se. (**d**) Raman spectra of Ti_3_AlC_2_ (MAX) and MXene nanosheets.

MXene and functionalized MXene were characterized using FT-IR spectroscopy (Figure 1a–c). Figure 1a displays the FTIR spectra of MXene, MXene-NH_2_, MXene-Se, and MXene-Se-DOX, while Figure 1b,c show the magnified FTIR spectra of MXene-NH_2_ and MXene-Se in order to clearly show the characteristic peaks of MXene-NH_2_ and MXene-Se nanosheets. For pristine MXene, the vibration peaks of the OH group were observed at 3427 cm^−1^ and 1620 cm^−1^, while a stretching vibration peak of Ti-O-Ti was observed at 540 cm^−1^ [26]. The characteristic peaks of MXene-NH_2_ were observed at 2967 cm^−1^ and 2886 cm^−1^, which correspond to the asymmetric and symmetric stretching vibrations of -CH-, respectively. The bending vibrations of N-H and C-N in MXene-NH_2_ were observed at 1527 and 1459 cm^−1^, respectively [27]. Furthermore, the characteristic peak at 3443 cm^−1^ for MXene-NH_2_ is derived from not only the O-H stretching vibration but also the N-H stretching vibration [28]. The aforementioned results proved that APTES was successfully grafted to the surface of the pristine MXene nanosheets (Figure 1b). After modification with DSeDPA, a new characteristic peak was observed at 1720 cm^−1^, which was attributed to the C=O group stretching vibration in the MXene-Se nanosheets. Additionally, the peaks at around 1579 cm^−1^ and at 1479 cm^−1^ correspond to N-H bending and C-N stretching vibrations, respectively, as shown in Figure 1c. All of these results indicated that the ROS-responsive crosslinker was conjugated to the surface of MXene. Meanwhile, the peaks at 3432 cm^−1^, 2928 cm^−1^, 1620/1477 cm^−1^, and 1087 cm^−1^ in the MXene-Se-DOX nanosheets are associated with the N-H stretching vibration, C-H stretching vibration, C=C ring stretching vibration, and C-O-C stretching vibration, respectively, which confirms the DOX conjugation on the surface of MXene. The FT-IR spectra revealed that MXene-Se-DOX nanosheets were successfully fabricated by facile condensation. To further verify the aforementioned results, the structural details of the Ti_3_AlC_2_ and MXene nanosheets were examined using Raman spectroscopy, as depicted in Figure 1d. The characteristic peaks of Ti_3_AlC_2_ observed at 268 cm^−1^, 412 cm^−1^, and 612 cm^−1^ were the result of the shear and longitudinal oscillations of titanium and aluminum atoms, which are in good agreement with those reported in the literature [29,30]. The feature peak at 268 cm^−1^ is attributed to vibrations of aluminum atoms, and this characteristic peak vanished after etching with LiF and HCl (minimally intensive layer delamination (MILD) method). An out-of-plane vibration peak of the Ti-C bond was observed at 208 cm^−1^ in the pristine MXene nanosheets. The scattering peak of MXene observed at 150 cm^−1^ after etching indicates the existence of oxidized Ti_3_C_2_. More importantly, the overlapping peaks of the D and G bands at 1365 cm^−1^ and 1570 cm^−1^ are attributed to graphitic carbon, which indicates the existence of amorphous carbon in MXene nanosheets [31]. These results show that ultrathin planar MXene nanosheets were successfully etched from the MAX phase using the MILD method and functionalized with DOX and DSeDPA.

### 2.2. Physicochemical Analysis

**Figure 2:**
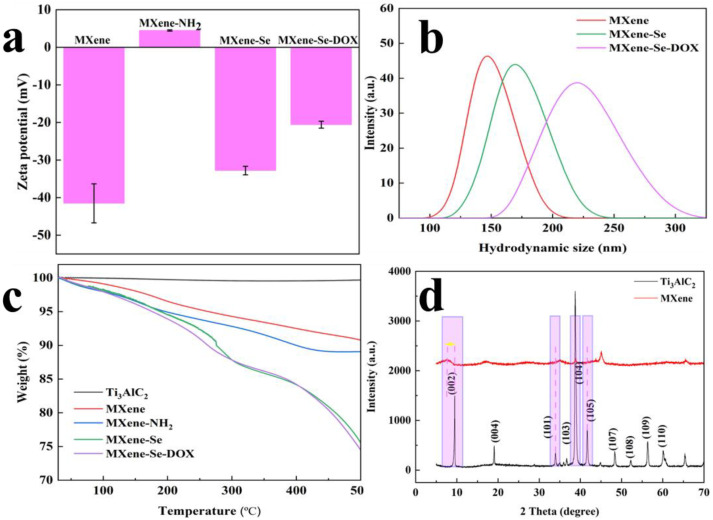
(**a**) Zeta-potential analysis of pristine MXene and functionalized MXene nanosheets. (**b**) Hydrodynamic diameters of MXene, MXene-Se, and MXene-Se-DOX nanosheets. (**c**) TGA curves of Ti_3_AlC_2_, MXene, MXene-NH_2_, MXene-Se, and MXene-Se-DOX nanosheets. (**d**) XRD patterns of Ti_3_AlC_2_ and MXene flakes.

The surface electronegativity of pristine MXene and functionalized MXene were measured using zeta potentials. As shown in Figure 2a, the pristine MXene surface exists at a high negative charge (−41.5 mV) in an aqueous solution, which is attributed to the OH groups on the surface of MXene [28,32]. After modification with APTES, the MXene-NH_2_ nanosheets exhibited a positive charge (4.48 mV), which is likely because of the protonated amine groups. Subsequently, the DSeDPA crosslinker was grafted to the surface of the MXene-NH_2_ nanosheets to form MXene-Se nanosheets, which exhibited a negative charge (−32.8 mV). This is possibly because of the presence of a carboxyl group. In this study, DOX was easily introduced to the MXene-Se surface between the amine and carboxyl groups [33]. The formed MXene-Se-DOX nanosheets exhibited a negative charge (−20.6 mV) in an aqueous solution, which is attributed to the abundant phenolic hydroxyl and alcohol hydroxyl groups. The serial changes in the zeta potentials from −41.5 mV to −20.6 mV demonstrate that APTES, DSeDPA, and DOX were successfully grafted to the MXene surface. Furthermore, the average hydrodynamic diameters of MXene, MXene-Se, and MXene-Se-DOX nanosheets in aqueous environments were measured to be approximately 142 nm, 164 nm, and 220 nm, respectively (Figure 2b). The increase in diameter is likely due to a series of surface modifications with APTES, DSeDPA, and DOX. The hydrodynamic diameter of MXene-Se-DOX has been reported to be approximately 200 nm, which is conducive to accumulating tumor sites owing to the EPR effect [34]. To further confirm the successful conjugation of APTES, DSeDPA, and DOX to the surface of MXene, we evaluated the weight loss from 30 °C to 500 °C using TGA (Figure 2c). Ti_3_AlC_2_ showed almost no change in weight loss over the entire temperature range up to 500 °C, demonstrating the excellent thermal stability of the MAX phase in the presence of N_2_. After etching, the MXene nanosheets exhibited an increasing trend of weight loss with increasing temperature, where the weight loss below 200 °C may be due to the evaporation of physisorbed water [35]. Further mass loss (8.9%) was subsequently observed, possibly due to the degradation of the functional groups (-OH, -F, and =O) of MXene. After APTES conjugation, MXene-NH_2_ showed a mass loss of approximately 10.6%, which is associated with the degradation of the functional groups in APTES. The functionalized MXene-Se showed a mass loss of 24% up to 500 °C owing to the decomposition of the DSeDPA crosslinker. Similarly, an additional mass loss of 25% was observed for the DOX-functionalized MXene nanosheets (MXene-Se-DOX), mainly because of the further degradation of DOX at high temperatures in a N_2_ environment. These data confirm the successful formation of MXene-Se-DOX nanosheets. The XRD patterns of the commercial Ti_3_AlC_2_ powder and MXene samples are shown in Figure 2d. The Ti_3_AlC_2_ powder exhibited characteristic Bragg diffraction peaks at (002), (004), (101), (103), (104), (105), (107), (108), (109), and (110), corresponding to 2θ values of 9.36°, 19.14°, 34.04°, 36.66°, 38.68°, 41.57°, 48.29°, 52.12°, 56.41°, and 60.16°, respectively. After etching, the main (104) peak associated with the Ti_3_AlC_2_ powder disappeared, whereas the (002) peak shifted to a lower 2θ angle from 9.4° (Ti_3_AlC_2_) to 7.6° (MXene) [32]. These results show that the aluminum atoms were successfully dislodged, and pristine MXene nanosheets were synthesized.

### 2.3. Morphology

**Figure 3:**
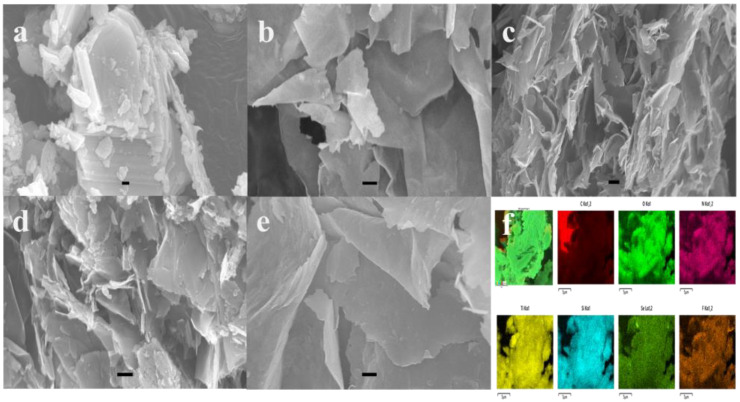
FE-SEM images of (**a**) Ti_3_AlC_2_, (**b**) MXene (Ti_3_C_2_), (**c**) MXene -NH_2_, (**d**) MXene-Se, and (**e**) MXene-Se-DOX and (**f**) EDX mapping of MXene-Se-DOX. The scale bars for (**a**–**e**) are 300 nm, while the scale bar for (**f**) is 5 μm.

**Figure 4:**
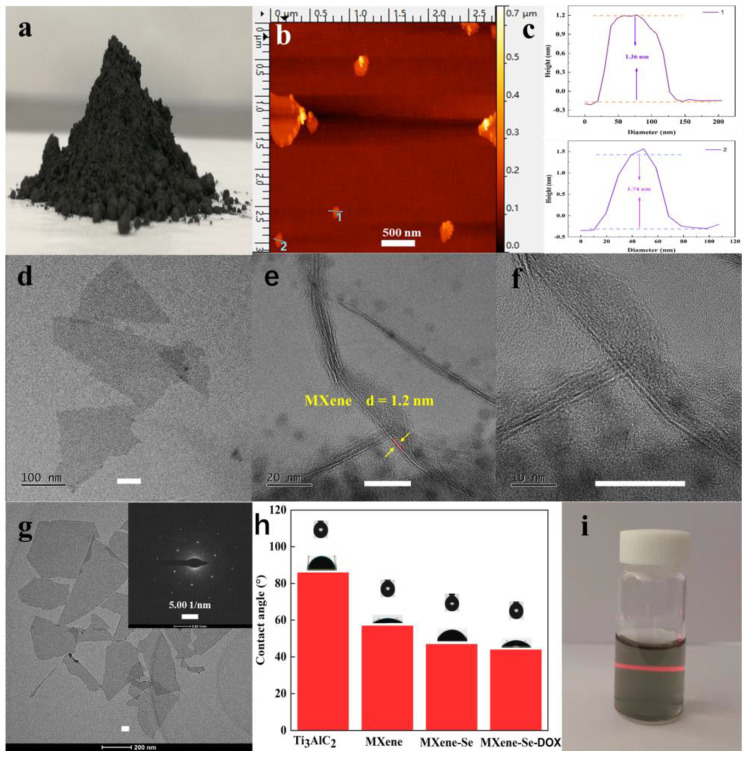
(**a**) Photograph of commercial Ti_3_AlC_2_ powder. (**b**) AFM image of MXene nanosheets. (**c**) Thickness and diameter of MXene nanosheets measured using AFM. (**d**) TEM image of ultrathin planar MXene nanosheets after exfoliation. (**e**,**f**) HR-TEM images of MXene nanosheets. (**g**) MXene flakes and the selected area electron diffraction (SAED) (inset). (**h**) Water contact angles for Ti_3_AlC_2_, MXene, MXene-Se, and MXene-Se-DOX nanosheets. (**i**) Tyndall effect detection in MXene nanosheets. The scale bars (white color) for (**b**,**d**,**g**), and (**e**,**f**) are 500, 50, and 20 nm, respectively, while the scale bar for SAED image (inset in the g image) is 5.00 nm^−1^.

The 2D planar nanostructure endows MXene with abundant anchoring sites and superior specific surface areas, which make it a promising delivery system for anticancer drugs [5,6]. Figure 3 shows the FE-SEM images of Ti_3_AlC_2,_ MXene_,_ MXene-NH_2,_ MXene-Se, and MXene-Se-DOX. The FE-SEM image of Ti_3_AlC_2_ exhibited layer-by-layer well-stacked particles, as shown in Figure 3a. After etching, an ultrathin planar flake was obtained, as depicted in Figure 3b, demonstrating that pristine MXene was successfully exfoliated from the MAX phase, which is in good agreement with the results obtained in a previous study [36]. After grafting with APTES, MXene-NH_2_ nanosheets with a smaller lateral size (below 200 nm) and high dispersibility were obtained, probably as a result of the interaction of the amine group, which further confirms the existence of APTES on the surface of MXene (Figure 3c). Compared with MXene-NH_2_ nanosheets, MXene-Se showed a morphology similar to that depicted in Figure 3d, indicating that the integrity of the nanosheets did not significantly affect the characteristic high specific surface area after functionalization with DSeDPA. Figure 3e shows an SEM image of MXene-Se-DOX with scattered flakes. These results confirm the successful formation of MXene-Se-DOX nanosheets. To further verify that DSeDPA and DOX were successfully grafted to the surface of MXene, we examined the elemental composition of MXene-Se-DOX nanosheets using EDX mapping, as shown in Figure 4f. The weight % of Ti, Si, and Se in the MXene-Se-DOX nanocarriers was found to be 18.72%, 3.29%, and 0.97%, respectively, which confirms the successful grafting of APTES, DSeDPA, and DOX compounds to MXene nanosheets. These results demonstrate the successful synthesis of MXene-Se-DOX nanosheets. Additionally, the EDS spectra of MXene-NH_2_ and MXene-Se nanosheets were examined, as depicted in Figures S4 and S5. Si and N elements were confirmed by the EDS spectrum in Figure S4, and the atomic % in the nanosheets was 3.77 and 0.4 %, respectively, which are attributed to the APTES chemical. Moreover, the EDS spectra revealed that the weight % of the Se element in MXene-Se nanosheets was 0.48 %, which demonstrates that the DSeDPA crosslinker was successfully conjugated to the surface of MXene. 

Figure 4a shows a photograph of the Ti_3_AlC_2_ powder. The AFM image shows that the nanostructure of MXene flakes from the Ti_3_AlC_2_ ceramic powder exhibits a typical 2D nanostructure with a lateral size of 70–200 nm and an approximate thickness of 1.5 nm (Figure 4b,c). Figure 4d shows a TEM image of pristine MXene nanosheets depicting highly dispersible MXene nanosheets with a lateral size of approximately 100 nm, which agrees with the AFM results. In addition, MXene chemical properties were examined using EDS mapping, as shown in Figure S6. From the EDS spectra, the weight % of C, Ti, O, and F in the MXene flakes was found to be 94.98%, 1.09%, 3.76%, and 0.17%, respectively. Furthermore, the crystal structure of MXene nanosheets was also investigated using high-resolution TEM (Figure 4e,f), which revealed a crystal structure with d _(002)_ = 1.2 nm. In the inset of Figure 4g, the selected area electron diffraction (SAED) pattern of the pristine MXene flakes shows a hexagonal symmetry structure and high crystallinity, which is also verified in the high-resolution TEM image. In addition, the water contact angle of the pristine MXene nanosheets was 57°, which diminishes immediately in a few seconds, revealing natural hydrophilicity. However, the water contact angle value of Ti_3_AlC_2_ nanosheets is 86° [37], which further demonstrates that the ultrathin planar MXene flakes were successfully exfoliated [38]. Furthermore, the water affinities of MXene-Se and MXene-Se-DOX nanosheets were evaluated. As shown in Figure 4h, the water contact angles of MXene-Se and MXene-Se-DOX nanosheets were 47° and 43°, respectively. This is because the MXene-Se flakes have hydrophilic carboxyl groups on their surfaces. In addition, the MXene-Se-DOX nanosheets also exhibited hydrophilicity, which is attributed to the hydrophilic groups of the alcohol hydroxyl and phenolic hydroxyl anchored on the surfaces of the nanosheets [39]. The contact angle results further demonstrate the grafting of DSeDPA and DOX to MXene flakes. Figure 4i shows the Tyndall effect of MXene nanosheets, which reveals a bright light path in aqueous solutions. This demonstrates that pristine MXene was successfully fabricated, and it exhibited excellent dispersibility in aqueous solutions.

### 2.4. XPS Spectra

**Figure 5:**
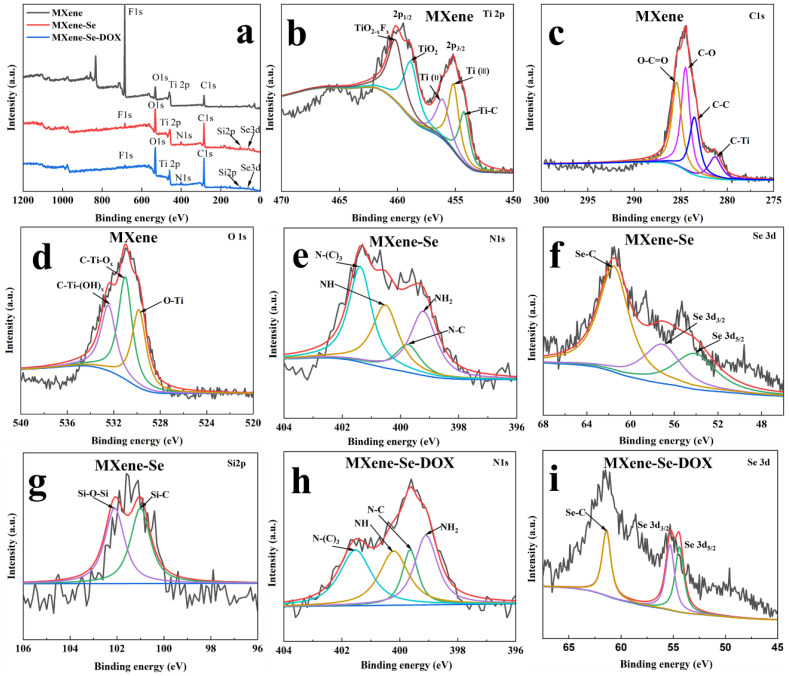
XPS spectra of MXene, MXene-Se, and MXene-Se-DOX nanosheets. (**a**) Survey scan spectrum of MXene, MXene-Se, and MXene-Se-DOX. (**b**–**d**) Ti 2p, C 1s, and O 1s spectra for MXene. (**e**–**g**) N 1s, Se 3d, and Si 2p spectra for MXene-Se. (**h**,**i**) N 1s and Se 3d spectra for MXene-Se-DOX.

The chemical composition, bonding form, and elemental valence of MXene-Se-DOX, MXene-Se, and pristine MXene were analyzed using X-ray photoelectron spectroscopy (XPS). The survey scan spectra of MXene-Se-DOX, MXene-Se, and MXene nanosheets show that the binding energies of Ti, C, O, Si, N, and Se are 454 eV, 284 eV, 532 eV, 102 eV, 400 eV, and 55 eV, respectively, as depicted in Figure 5a. The high-resolution XPS spectra of Ti 2p, C 1s, and O 1s of MXene are shown in Figure 5b–d, respectively. Ti 2p3/2 was observed at 454.2 eV, 456 eV, 455.1 eV, 458.8 eV, and 459.6 eV. The Ti 2p peaks correspond to Ti-C, Ti(II), and Ti(III), which are ascribed to Ti bonded to C at 454.2 eV and Ti atoms bonded to -O or -OH at 456 eV and 455.1 eV, respectively. The Ti 2p XPS spectra are mainly composed of Ti-C, Ti(II), and Ti(III) species. Ti 2p3/2 was observed at 459.6 eV and 458.8 eV, indicating that Ti_3_C_2_ was partially oxidized to form TiO_2_ and TiO_2-x_F_x_ on the surface (Figure 5b) [40]. C1s was observed at 281.2 eV, 283.5 eV, 284.5 eV, and 285.4 eV, corresponding to C–Ti, C–C/C–H, C–O, and O–C=O bonds [5,6,41], respectively, as depicted in Figure 5c. Generally, O 1s was deconvoluted into three peaks at 532.5 eV, 531.1 eV, and 529.9 eV, which are attributed to C-Ti-(OH)_X_, C-Ti-O_X_, and O-Ti bonds, respectively, as shown in Figure 5d [42]. The high-resolution XPS spectra of the N 1s, Se 3d, and Si 2p core levels of MXene-Se after their functionalization with DSeDPA are shown in Figure 5e–g, respectively. As shown in Figure 5e, the XPS spectrum of N1s with binding energy at 399.6 eV corresponds to the N-C bond, whereas the N1s spectrum corresponds to the primary, secondary, and tertiary amine peaks at 399.1 eV, 400.2 eV, and 401.4 eV, respectively [43], demonstrating the successful functionalization of the ROS-responsive DSeDPA on the surface of MXene. For the Se 3d spectrum of the MXene-Se flakes, two peaks associated with the Se 3d_3/2_ and 3d_5/2_ orbitals are shown in Figure 5f, which correspond to 55.3 eV and 54.4 eV, respectively [44,45]. The Se 3d spectrum of the MXene-Se flakes was observed at 61.4 eV, which is attributed to the Si-C bond. In addition, the high-resolution Si 2p XPS spectrum was split into two peaks at 102.2 eV and 101.1 eV, which are consistent with Si-O-Si and Si-C, respectively (Figure 5g). In the MXene-Se nanosheets, the XPS spectra of N 1s, Si 2p, and Se 3d nanosheets were observed, which demonstrates that the ROS-responsive crosslinker was successfully introduced to the MXene surface. Importantly, the XPS spectra and elemental contents of N 1s and Se 3d in MXene-Se-DOX nanosheets were detected, as presented in Figure 5h,i and Table 1. The N content in MXene-Se-DOX nanosheets increased from 3.49% to 3.57% compared with the MXene-Se nanostructure, possibly because of DOX containing the nitrogen element. These results further confirmed that the MXene-Se-DOX nanosheets were successfully synthesized. Furthermore, the XPS spectra of Ti 2p and C1s for MXene-Se and Ti 2p and C1s, Se 3d, and Si 2p for MXene-Se-DOX were also detected, as shown in Figure S7.

### 2.5. In Vitro Antibacterial Activity 

**Figure 6:**
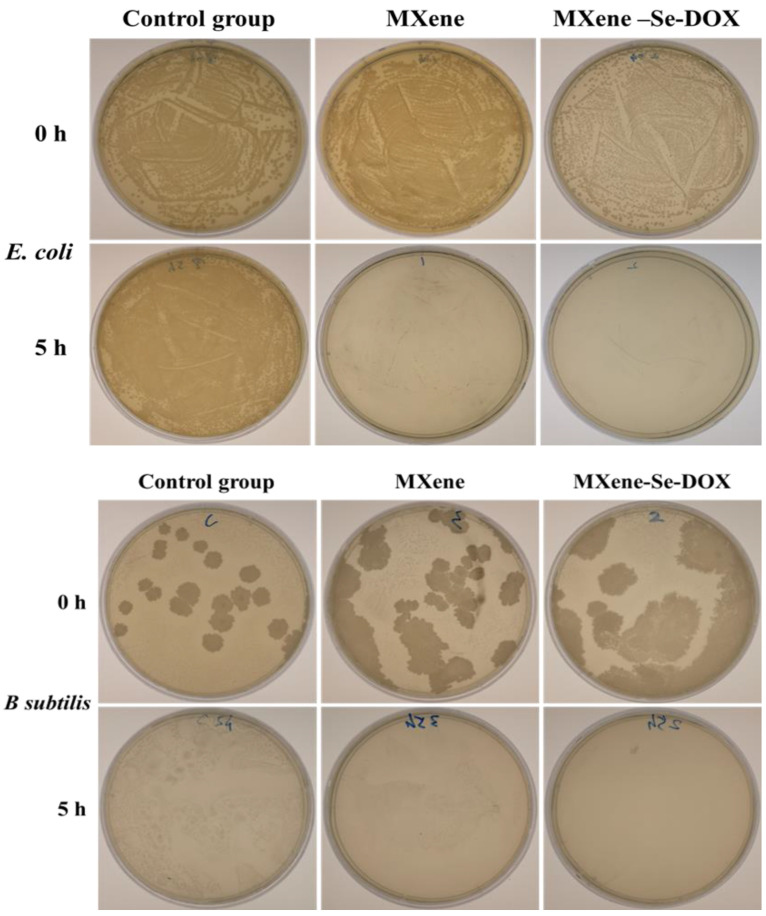
Antibacterial activities in an aqueous solution after 5 h treatment: bacterial suspensions in PBS 7.2 solution (0.01 mM) acted as control. The MXenen and MXene-Se-DOX nanosheets with a concentration of 6.085 mg/mL were recultivated onto *E. coli* and *B. subtilis* bacteria, as shown in the photographs of agar plates. All sample analyses were performed in triplicate.

The antibacterial activity of nanomaterials has also recently attracted research interest because bacterial pathogens can cause many serious diseases after infection [46]. It is well-known that pristine MXene (prepared using the MILD method) exhibits antibacterial activity against Gram-negative and Gram-positive bacteria. In this study, we examined the antibacterial activity of functionalized MXene nanosheets (MXene-Se-DOX). To determine the antibacterial effects of MXene and MXene-Se-DOX nanosheets, we examined the inhibitory effects of both nanomaterials against *E. coli* and *B. subtilis*. First, the microbe was activated in Luria–Bertani medium and diluted 200-fold. The supernatant was then resuspended in an equal volume of a nanosheet solution. The antibacterial ability of the nanosheets was evaluated using the colony-counting method. The control and bacterial cell groups were monitored after treatment for 5 h with the same concentration of 6.085 mg/mL nanosheets, as shown in the photographs of the agar plates in Figure 6. Many colonies were observed after 0 h of treatment, which indicates that the bacteria were well activated in both the control and treated groups. However, the treated groups presented high growth inhibition abilities against bacterial cells of both Gram (−) *E. coli* and Gram (+) *B. subtilis*. The control group exhibited a negligible antimicrobial effect after treatment for 5 h. In this study, the MXene nanosheets exhibited a high percentage of growth inhibition against *E. coli* and *B. subtilis*, which is associated with the delaminated MXene nanosheets with sharp edges and a tendency to adsorb to bacterial cell surfaces and cause membrane damage to bacterial cells. It was reported that the smallest MXene nanosheets penetrate microbial cells and react with molecules in the cytoplasm, further disrupting the cell structure [15]. The MXene-Se-DOX nanosheets exhibited a higher antibacterial efficiency compared with the pristine MXene nanosheets, which is likely attributed to the toxicity of DOX released in the bacterial microenvironment. The number of bacteria in the photograph of the MXene-Se-DOX group is negligible, which demonstrates that the properties of MXene functionalized with DSeDPA and DOX were not significantly changed. These results indicate that MXene-Se-DOX nanosheets have a remarkable inhibitory effect against *E. coli* and *B. subtilis.* All sample analyses were performed in triplicate.

### 2.6. In Vitro Drug Release Profile 

**Figure 7:**
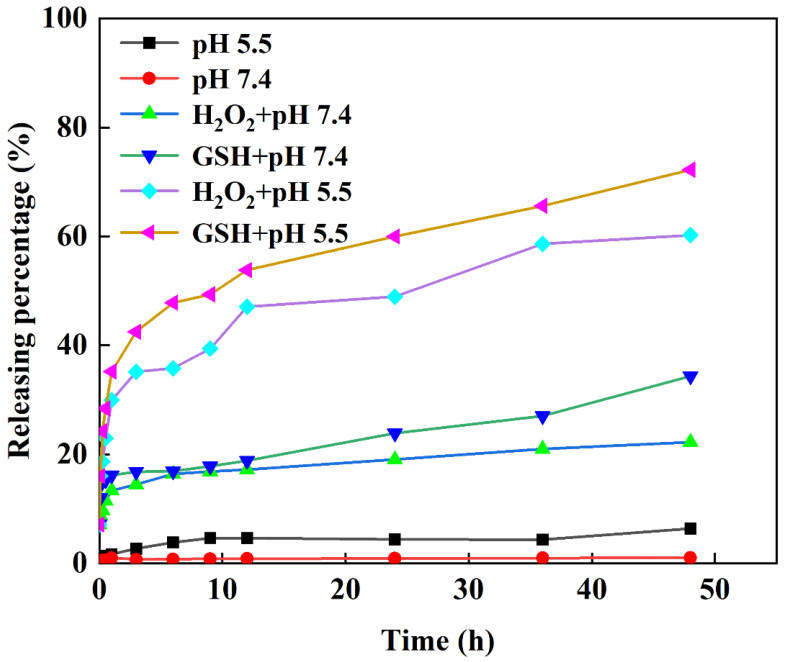
DOX release percentage from MXene-Se-DOX nanocarriers in different media.

The loading capacity and encapsulation efficiency of DOX for the MXene-Se-DOX nanocarriers were estimated at 17.95% and 41.66%, respectively, using Equations (2) and (3) with the aid of a calibration curve. The percentage of DOX released was determined using Equation (4) The DOX release performance of MXene-Se-DOX nanosheets in diverse media is shown in Figure 7. A release percentage of 1.09% was observed under physiological pH (7.4) conditions after 48 h, which demonstrates that the MXene-Se-DOX nanocarriers could efficiently prevent the premature release of DOX during blood circulation. The DOX release rate of MXene-Se-DOX nanocarriers is still low in weak acidic conditions (pH 5.5) compared with neutral conditions. This is possibly because DOX was covalently bonded to the MXene surface [47], effectively limiting the premature release of DOX. To examine the ROS-responsive behavior of MXene-based nanocarriers, we further evaluated the DOX release profiles from MXene-Se-DOX nanocarriers in H_2_O_2_ or GSH solutions with different pH (5.5 and 7.4) levels. At pH 7.4, the cumulative release rate of the nanocarriers was 22.3% under H_2_O_2_ conditions after 48 h. However, a slightly higher release percentage (34.3%) was observed in the presence of GSH after 48 h. This is likely because of the cleavage of the diselenide bond from 3,3′-diselanediyldipropionic acid, which is more sensitive to the GSH environment. We examined the drug release rate under physiological conditions (pH 7.4) and determined the DOX release performance in a tumor microenvironment. As expected, the MXene-Se-DOX nanocarriers exhibited a higher DOX release percentage (60.2%) in a H_2_O_2_ solution at pH 5.5 after 48 h. The highest amount (72%) of DOX was released from MXene-Se-DOX under GSH conditions at pH 5.5 after 48 h because the diselenide bond was easily cleaved by reducing agents [40,48], which offered an opportunity to detach the DOX molecules. The release performance results of MXene-Se-DOX nanocarriers demonstrated the ability to control release as well as good ROS-responsive and pH-responsive properties, indicating a dual (both ROS- and pH-) responsive behavior.

### 2.7. Drug Release Kinetics 

The release kinetics of DOX from the MXene-Se-DOX nanocarriers were investigated using the Korsmeyer–Peppas and Higuchi models. The parameters of the release mechanism from the Korsmeyer–Peppas model are listed in Table 2 [49]. The Korsmeyer–Peppas empirical equation is as follows [43]:

Korsmeyer-Peppas: M_t_/M_∞_ = kt_n_ (1)

where M_t_/M_∞_ is the drug release percentage at time t, k_p_ is the rate constant, and n is the release exponent. 

Obviously, the release mechanism of DOX from MXene-based nanocarriers fitted the Korsmeyer–Peppas model better compared with the Higuchi model, indicating a higher correlation coefficient (R^2^). Furthermore, the release exponent (n) values were 0.271, 0.096, 0.143, 0.159, 0.209, and 0.201 at pH 5.5, pH 7.4, pH 7.4 + H_2_O_2_, pH 7.4 + GSH, pH 5.5 + H_2_O_2_, and pH 5.5 + GSH, respectively. These results show that the DOX release exponent values are less than 0.43 in different release media, which indicates that the release kinetics from MXene-Se-DOX nanosheets followed the Fickian diffusion mechanism [50,51,52]. These results indicate that Fickian diffusion plays a major role in the release process. It should be noted that smaller R² values were obtained with the Higuchi models [53,54], confirming that they are unsuitable release mechanisms for the studied MXene-Se-DOX nanocarriers (Table S1).

## 3. Materials and Method

### 3.1. Materials

Commercial Ti_3_AlC_2_ powder (MAX phase, ≥99%, ~400 mesh) was obtained from Jilin Yiyi Technology Co. Ltd. (Jilin, China). Sigma-Aldrich (Saint Louis, MO, USA) provided 3-aminopropyltriethoxysilane (APTES, 98%), toluene (anhydrous 99.8%), lithium fluoride (99.99%), and doxorubicin (DOX, 99% HPLC). We purchased 1-(3-dimethylaminopropyl)-3-ethylcarbodiimide hydrochloride (EDC, >98.0%) from TCI Company (Tokyo, Japan). Tetrahydrofuran (THF anhydrous ≥ 99.9%), selenium powder (99.99%), sodium borohydride (99%), 3-bromopropionic acid (97%), and 1-hydroxy-2,5-pyrrolidinedione (NHS, 98.0%) were obtained from Sigma-Aldrich Company (Saint Louis, MO, USA). Hydrogen peroxide (H_2_O_2_) (30%) and glutathione (GSH) (pharmaceutical secondary standard) were obtained from Sigma-Aldrich (Saint Louis, MO, USA). Phosphate-buffered saline (PBS, pH 7.4) was obtained from Welgene (Gyeongsan, Republic of Korea). Hydrochloric acid was obtained from Daejung Chemical Co. Ltd. (Siheung, Republic of Korea).

### 3.2. Characterization 

Fourier transform infrared (FT-IR, FTIR 4100) spectra (JASCO Co., Tokyo, Japan) were obtained using KBr pellets in the frequency range 4000–400 cm^−1^. X-ray diffraction (XRD, Bruker AXS) (Billerica, MA, USA) was performed using Cu-Kα radiation, and the data were collected in wide-angle ranges from 5° to 70° 2θ. The lateral size and thickness of the nanosheets were examined using atomic force microscopy (AFM; Park System NX10) (Park System, Suwon, Republic of Korea). The crystal structure and morphology of the nanosheets were characterized at an accelerating voltage of 200 kV using high-resolution transmission electron microscopy (HR-TEM, JEOL 2010) (JEOL Ltd., Tokyo, Japan). The Raman spectra (JASCO, NRS-5000 Series, Tokyo, Japan) were obtained using a visible laser at 532 nm. Field-emission scanning electron microscopy (FE-SEM, JEOL 6400) (JEOL Ltd., Tokyo, Japan) images were collected at an operating voltage of 20 kV. The drug release rate and drug-loading capacity of the samples were measured using ultraviolet–visible (UV-Vis) spectrophotometry (UV–1650, Shimadzu, Kyoto, Japan). The surface electronegativity of the nanosheets was determined using a zeta-potential analyzer (Zetasizer Nano-ZS, Malvern, UK). The thermal stability of the products was evaluated using thermogravimetric analysis (TGA, Q50 V6.2, Build 187, TA Instruments, New Castle, DE, USA) in a N_2_ atmosphere from 30 °C to 500 °C at a heating rate of 10 °C/min. The molecular structure and chemical composition of DSeDPA were analyzed using nuclear magnetic resonance (NMR) spectroscopy (Bruker (300 MHz)) (Bruker Co., Billerica, MA, USA) using DMSO-d_6_ as the solvent. The contact angle of the nanosheets was measured using a contact angle analyzer (SEO Pheonix 300, Suwon, Republic of Korea). The hydrodynamic diameter of the nanosheets was determined by dynamic light scattering (DLS, Zetasizer NANO-S90, Malvern, UK). A chemical composition analysis of the as-grafted and pristine MXenes was performed using X-ray photoelectron spectroscopy (XPS, VG Scientific (U.K.), Multi Lab.) with Al Kα radiation.

### 3.3. Synthesis of MXene Nanosheets

**Figure 8:**
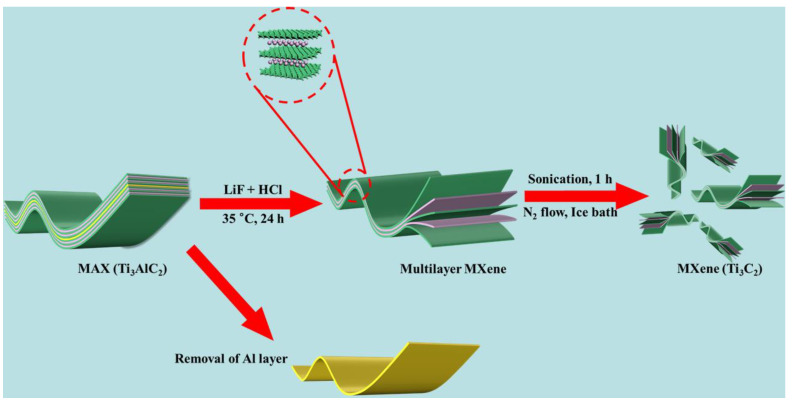
Schematic diagram of the synthesis procedure of ultrathin 2D MXene nanosheets.

First, LiF (6.4 g) was added to a Teflon bottle containing 9 M HCl (80 mL) and stirred for 30 min to completely dissolve the LiF [55]. Then, Ti_3_AlC_2_ powder (4 g) was slowly added to this solution. It should be noted that the slow addition of Ti_3_AlC_2_ powder prevents the sputtering of hydrofluoric acid. The Teflon bottle was then immersed in an oil bath at 35 °C for 24 h. The Al layer was successfully etched after 24 h. The product was purified by centrifugation using deionized water until the pH of the supernatant solution reached 6. The precipitate was collected by centrifugation at 3500 rpm for 30 min. The collected precipitate was dispersed in 200 mL of water and sonicated for 1 h in an ice bath under flowing Ar. To obtain pure MXene, we centrifuged the mixture at 3500 rpm for 30 min and collected and stored the supernatant in a refrigerator at 4 °C for future use. The etching process for Ti_3_AlC_2_ is shown in Figure 8 (yield: 46%).

### 3.4. Synthesis of MXene-NH_2_ Nanosheets

MXene (1 g) was dispersed in toluene (60 mL) and stirred at room temperature for 30 min. Then, APTES (2 mL) was injected into the solution, which was heated to 105 °C in a condensate reflux tube for 12 h. MXene-NH_2_ was purified six times with water and ethanol by centrifugation to remove excess APTES. Finally, the product was vacuum freeze-dried for 48 h [56].

### 3.5. Synthesis of 3,3′-Diselanediyldipropionic Acid

The synthesis procedure for 3,3′-diselanediyldipropionic acid is shown in Figure S1. Selenium powder (0.635 g) and sodium borohydride (0.3057 g) were dispersed in ethyl alcohol (40 mL) in the presence of nitrogen for 1 h at room temperature [38]. 3-Bromopropioric acid (1.2616 g) was dissolved in ethyl alcohol (8 mL). Subsequently, the 3-bromopropioric acid solution was injected dropwise into the above solution under a nitrogen atmosphere and stirred at room temperature for 24 h. The reaction was quenched by adding ethyl acetate, and the product was precipitated. A pale-yellow precipitate was obtained, washed with water several times, and dried in an oven for 48 h (65 % yield). Furthermore, the structure of the DSeDPA crosslinker was determined using NMR spectroscopy (Figure S2).

^1^H-NMR (600 MHz, DMSO) δ 2.69 (a; 16H; -OC(=O)CH_2_CH_2_SeSeCH_2_CH2C(=O)O- of DSeDPA), δ 3.03 (b; 16H; -OC(=O)CH_2_CH_2_SeSeCH_2_CH_2_C(=O)O- of DSeDPA). ^13^C NMR (600 MHz, DMSO) δ 173.57 (c; -OC(=O)CH_2_CH_2_SeSeCH_2_CH_2_C(=O)O- of DSeDPA), δ 35.90 (a; -OC(=O)CH_2_CH_2_SeSeCH_2_CH_2_C(=O)O- of DSeDPA), δ 24.42 (b; -OC(=O)CH_2_CH_2_SeSeCH_2_CH_2_C(=O)O- of DSeDPA).

### 3.6. Synthesis of MXene-Se Nanosheets

To introduce the ROS-responsive crosslinker, we added the aqueous solution of MXene-NH_2_ (25 mL, 2 mg/mL) to the aqueous solution of DSeDPA (25 mL, 2 mg/mL) containing EDC (50 mM, 0.3 mL) and NHS (50 mM, 0.25 mL) for 48 h at room temperature. Subsequently, the MXene-Se nanosheets were washed with distilled water four times and freeze-dried for two days [57].

### 3.7. Synthesis of MXene-Se-DOX Nanosheets

Doxorubicin hydrochloride (30 mg) was desalted using 2 mL of DMSO containing triethylamine (50 μL) in a dark environment. The DMSO solution of MXene-Se (20 mL, 3 mg/mL) was activated with EDC (60 mg) and NHS (40 mg) for 1 h. Next, the DOX solution was injected into the mixture and allowed to react for another two days at room temperature. Excess chemicals were removed using water, washed three times by centrifugation (5 min, 4000 rpm), and freeze-dried for 48 h.

### 3.8. Antibacterial Activity 

The bacteriostatic performance of the samples was evaluated using the standard colony-counting method [28]. The microbes were activated in Luria–Bertani (LB) medium at 30 °C. The microbial suspension was diluted 200-fold and resuspended in an equal volume of 0.01 mM phosphate buffer solution/(6.085 mg/mL MXene or MXene-Se-DOX). Subsequently, the diluted microbial liquid (0 h, 5 h) was spread evenly on the solid LB medium. Finally, the solid media were cultured at 30 °C for 10 h, and the viable numbers of microbial colonies were counted by visual observation. All sample analyses were performed in triplicate. 

### 3.9. Loading Capacity and Drug Release Study

MXene-Se-DOX (10 mg) was dispersed in H_2_O_2_ /PBS (1.5/1.0 *v*/*v*) solution (5 mL) to release the doxorubicin molecule. The solution was shaken for 12 h. The supernatant was collected after centrifugation (4000 rpm, 15 min) for UV measurement at 485 nm. The concentration of DOX was calculated using its calibration curve in a PBS solution (Figure S3). 

The loading capacity (LC) and encapsulation efficiency (EE) were calculated using the following equations [44].

(2) $$\mathrm{LC}\;(\%)=\frac{\mathrm{Weight}\;\mathrm{of}\;\mathrm{loaded}\;\mathrm{DOX}\;\mathrm{in}\;\mathrm{nanoparticles}\;}{\mathrm{Weight}\;\mathrm{of}\;\mathrm{nanoparticles}}\times 100$$

(3) $$\mathrm{EE}\;(\%)=\frac{\;\mathrm{Weight}\;\mathrm{of}\;\mathrm{loaded}\;\mathrm{DOX}\;\mathrm{in}\;\mathrm{nanoparticles}}{\mathrm{Weight}\;\mathrm{of}\;\mathrm{DOX}\;\mathrm{in}\;\mathrm{feed}}\times 100$$

The DOX release profiles of the MXene-Se-DOX nanosheets were obtained in different media using a shaker (120 rpm) at 37 °C. MXene-Se-DOX nanosheets (3 mg) were initially placed in a dialysis bag with a 12,000 Da molecular weight cutoff containing different media (PBS, PBS + GSH, PBS + H_2_O_2_, PBS 5.5, PBS 5.5 + GSH, and PBS 5.5 + H_2_O_2_) solutions (5 mL). Subsequently, the dialysis sacks were immersed in a buffer solution. At a predetermined time, 3 mL of buffer solution was removed and replenished with the same volume of fresh buffer solution. The absorbance of DOX was monitored using UV–Vis spectroscopy at 485 nm. 

The percentage of DOX released was calculated using the following equation [58].

% Rt =C_t_·V_1_ + V_2_· (C _t−1_ + C _t−2_ + · · · + C_0_)/W_0_·L × 100% (4)

where C_t_ denotes the drug concentration at time interval t, C_t−1_ and C_t−2_ are the drug concentrations prior to time interval t (C_0_ = 0), V_1_ is the total volume, and V_2_ is the volume extracted. W_0_ represents the initial weight of the DOX-loaded MXene-Se-DOX, and L is the loading content (obtained from Equation (2)). All sample analyses were performed in triplicate. 

## 4. Conclusions

In summary, a novel MXene-Se-DOX nanocarrier with a lateral size and thickness of approximately 200 nm and 1.5 nm, respectively, was designed and investigated for its drug delivery behavior and antibacterial activity against Gram (−) *E. coli* and Gram (+) *B. subtilis* in vitro. The MILD etching process was used to obtain well-dispersed, pristine MXene nanoflakes to meet the requirements of biomedical applications. Afterward, the MXene-Se-DOX nanocarrier was prepared via the introduction of DSeDPA and doxorubicin on the surface of MXene nanosheets. Compared to the MAX phase, the contact angle of the original MXene nanosheets dropped dramatically from approximately 86 °C to 35 °C, which demonstrates that MXene nanosheets are hydrophilic materials. MXene-based nanosheets (MXene-Se-DOX) exhibited significant stimulus-response behaviors and the controlled release of the loaded drug in response to certain stimuli, such as high concentrations of H_2_O_2_ or GSH/PBS solution at two different pH levels (5.5 and 7.4). Importantly, MXene-Se-DOX nanosheets at a concentration of 6.085 mg/mL in an aqueous solution exhibited high antibacterial activity. It is expected that MXene-Se-DOX nanosheets will be a promising potential drug delivery platform for tumor therapy and a new method for antibacterial applications. We also hope that MXene-Se-DOX will further broaden the application of MXene-based nanoplatforms in different fields, especially in the biomedical area.

## Figures and Tables

**Table 1:** Atomic concentrations of elements on the surfaces of MXene, MXene-Se, and MXene-Se-DOX nanosheets.

Sample	Element At. %						
	Ti	C	O	F	N	Si	Se
MXene	7.36	28.23	12.58	50.59	-	-	-
MXene-Se	7.68	55.42	0.73	1.83	3.49	4.56	0.73
MXene-Se-DOX	6.54	54.09	28.62	1.44	3.57	4.34	0.29

**Table 2:** In vitro DOX release mechanism analysis based on the Korsmeyer–Peppas model.

Sample Code	Release Medium	Korsmeyer–Peppas ^a^		
		n	k_p_	R^2^
	pH 5.5	0.271	2.075	0.898
	pH 7.4	0.096	0.713	0.937
	pH 7.4 + H_2_O_2_	0.143	12.51	0.974
MXene-Se-DOX	pH 7.4 + GSH	0.159	15.16	0.913
	pH 5.5 + H_2_O_2_	0.209	26.67	0.964
	pH 5.5 + GSH	0.201	32.52	0.979

^a^ k_p_ is the release rate constant, n is the release exponent, and R^2^ is the correlation coefficient.

## Data Availability

Not applicable.

## Supplementary Materials

The following supporting information can be downloaded at: https://www.mdpi.com/article/10.3390/ijms232314925/s1.
