# Supplementary material for: Dual (pH- and ROS-) Responsive Antibacterial MXene-Based Nanocarrier for Drug Delivery
Source: Int J Mol Sci. 2022 Nov 29;23(23):14925. doi: 10.3390/ijms232314925 (PMC9739462; doi:10.3390/ijms232314925)
Supplement: Supplementary file 1 [file ijms-23-14925-s001.zip › ijms-2008852-supplementary.pdf]

Supplentary Materials

A Dual (pH- and ROS-) Responsive Antibacterial MXene-based Nanocarrier for Drug Delivery

Wei-Jin Zhang [1], Shuwei Li [2], Yong-Zhu Yan[1], Sung Soo Park [3], Anandhu Mohan [4], Ildoo Chung [1], Suk-kyun Ahn [1], Jung Rae Kim [2], Chang-Sik Ha [1*]

[1]Department of Polymer Science and Engineering, School of Chemical Engineering, Pusan National University, Busan 46241, Republic of Korea.

[2]School of Chemical Engineering, Pusan National University, Busan, 46241, Republic of Korea.

[3]Division of Advanced Materials Engineering, Dong-Eui University, Busan 47340, Republic of Korea.

[4]Department of Industrial and Environmental Engineering, Gachon University, Seongnam 13120, Republic of Korea.

* Correspondence: csha@pnu.edu (Chang-Sik Ha)

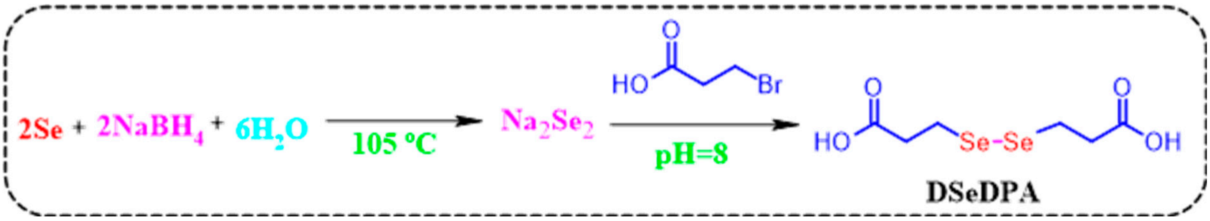

Figure S1 The synthesis procedure of DSeDPA.

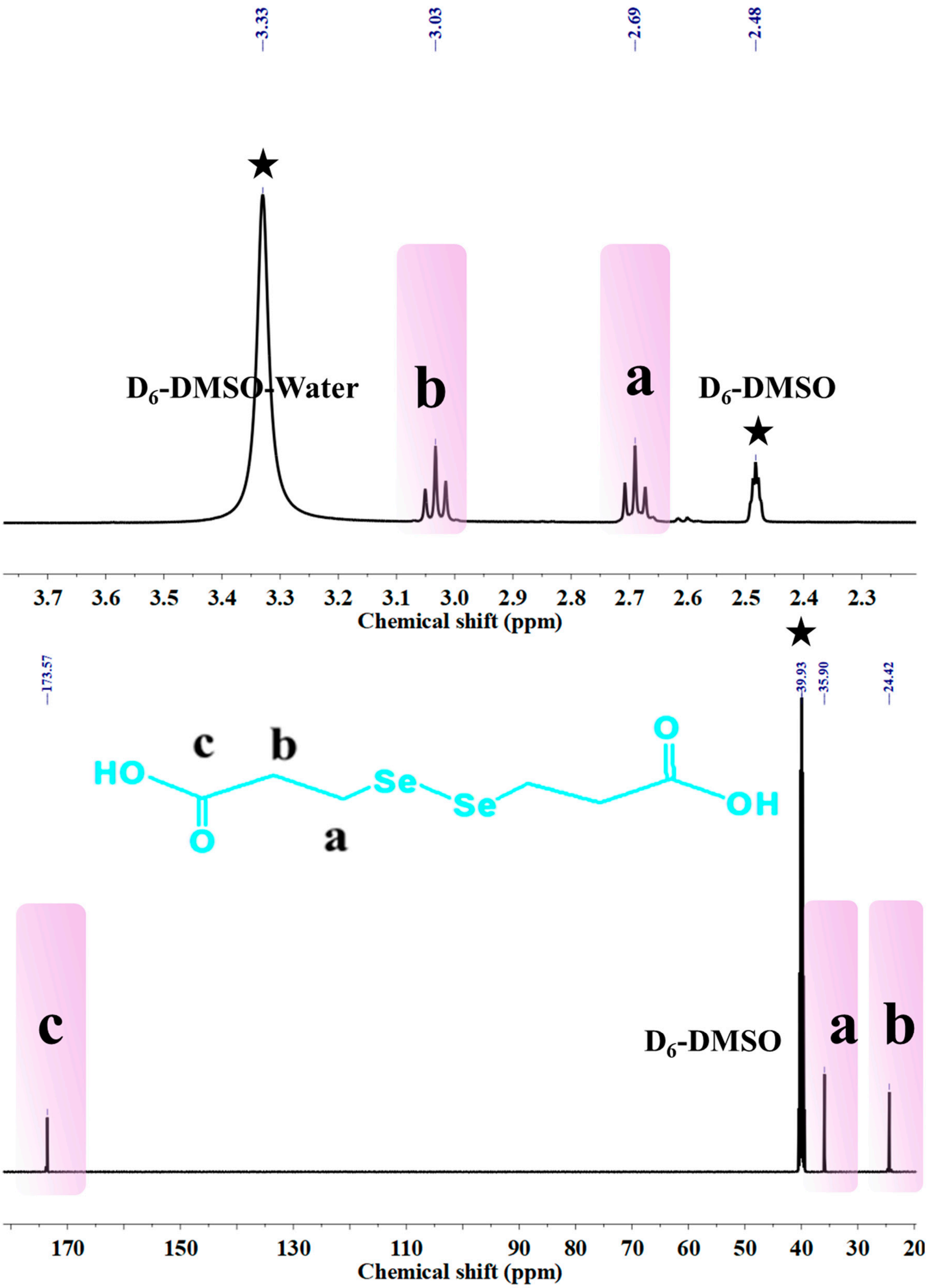

Figure S2 ^{1}H and ^{13}C NMR spectra of 3,3′-diselanediyldipropionic acid cross-linker in DMSO-d_6.

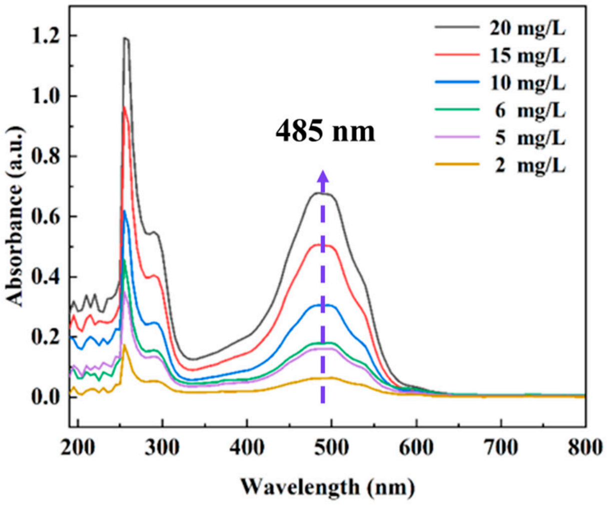

Figure S3 The calibration curve of DOX in PBS 7.4 solution.

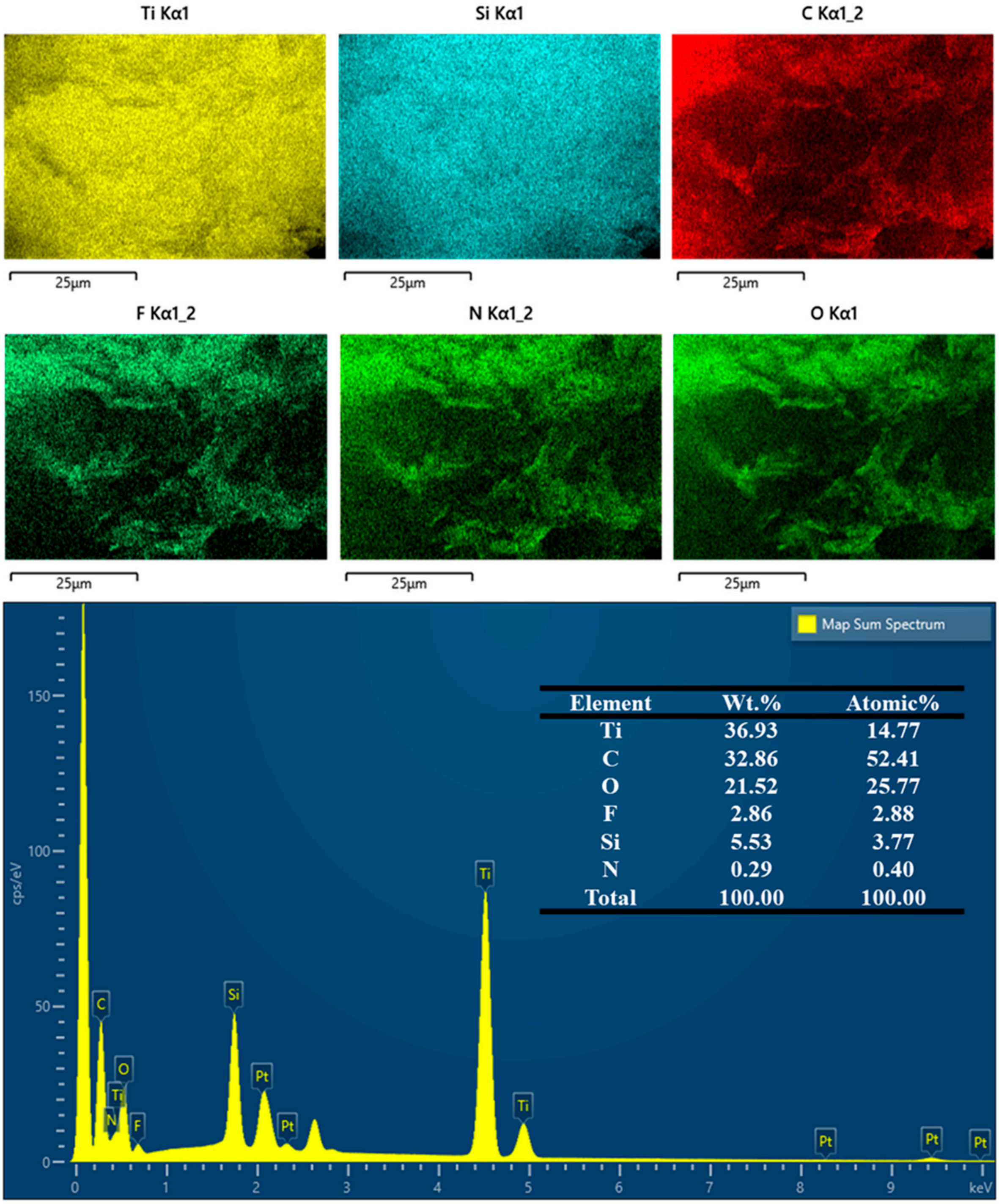

Element	Wt.%	Atomic%
Ti	36.93	14.77
C	32.86	52.41
O	21.52	25.77
F	2.86	2.88
Si	5.53	3.77
N	0.29	0.40
Total	100.00	100.00

Figure S4 The EDX (mapping) of MXene-NH_2 nanosheets.

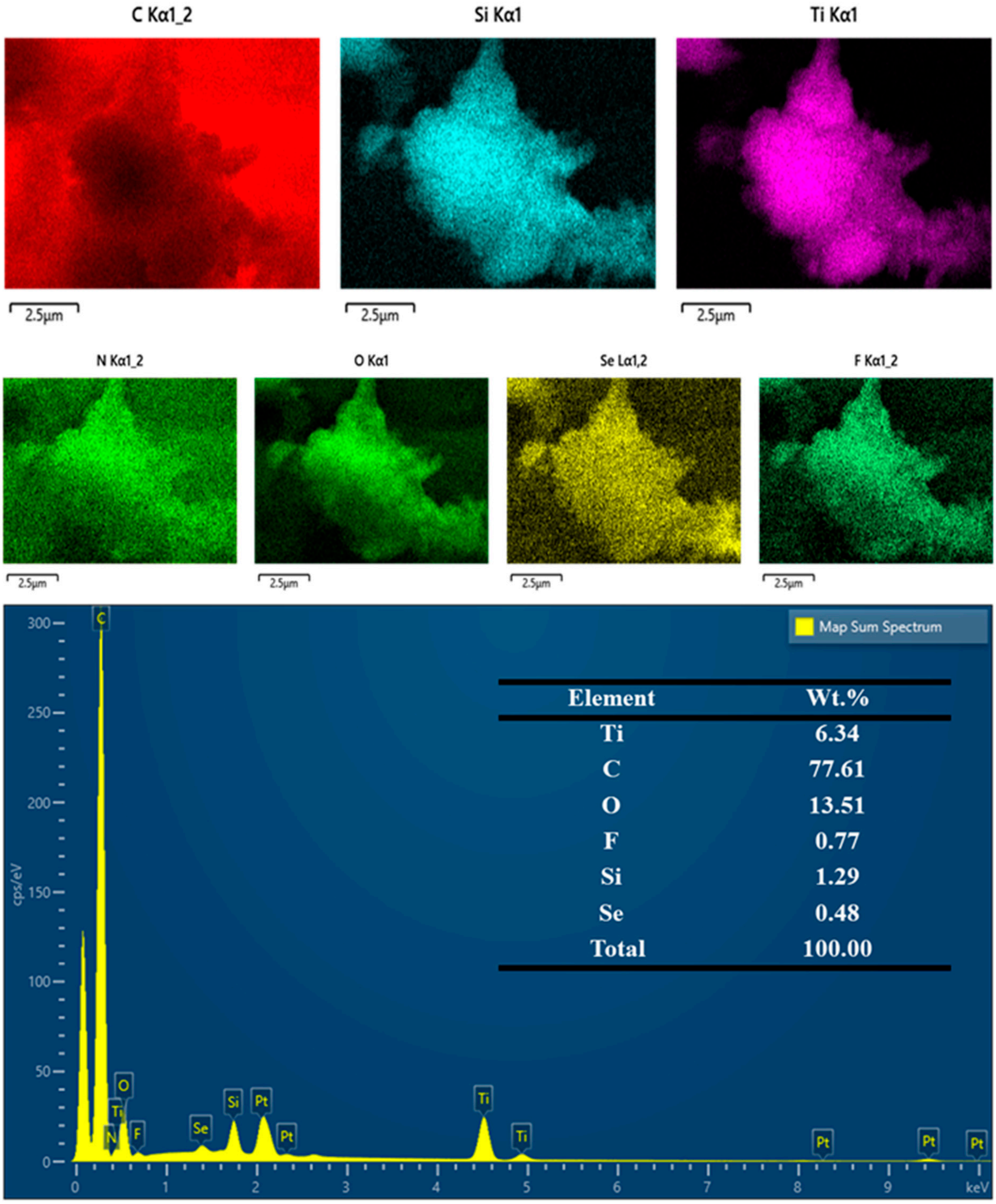

Figure S5 The EDX (mapping) of MXene-Se nanosheets.

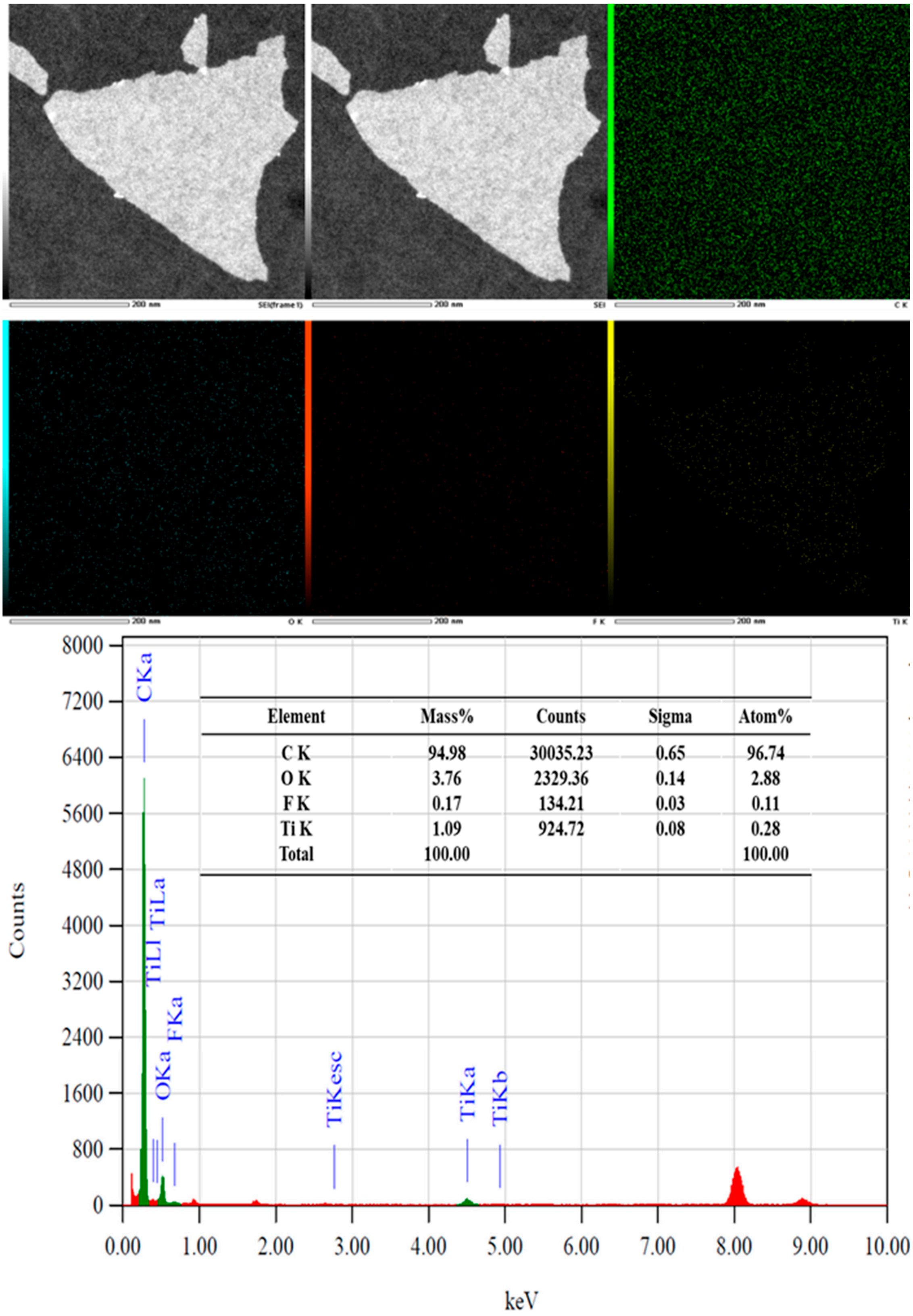

Element	Mass%	Counts	Sigma	Atom%
C K	94.98	30035.23	0.65	96.74
O K	3.76	2329.36	0.14	2.88
F K	0.17	134.21	0.03	0.11
Ti K	1.09	924.72	0.08	0.28
Total	100.00			100.00

Figure S6 The EDX (mapping) of MXene nanosheets.

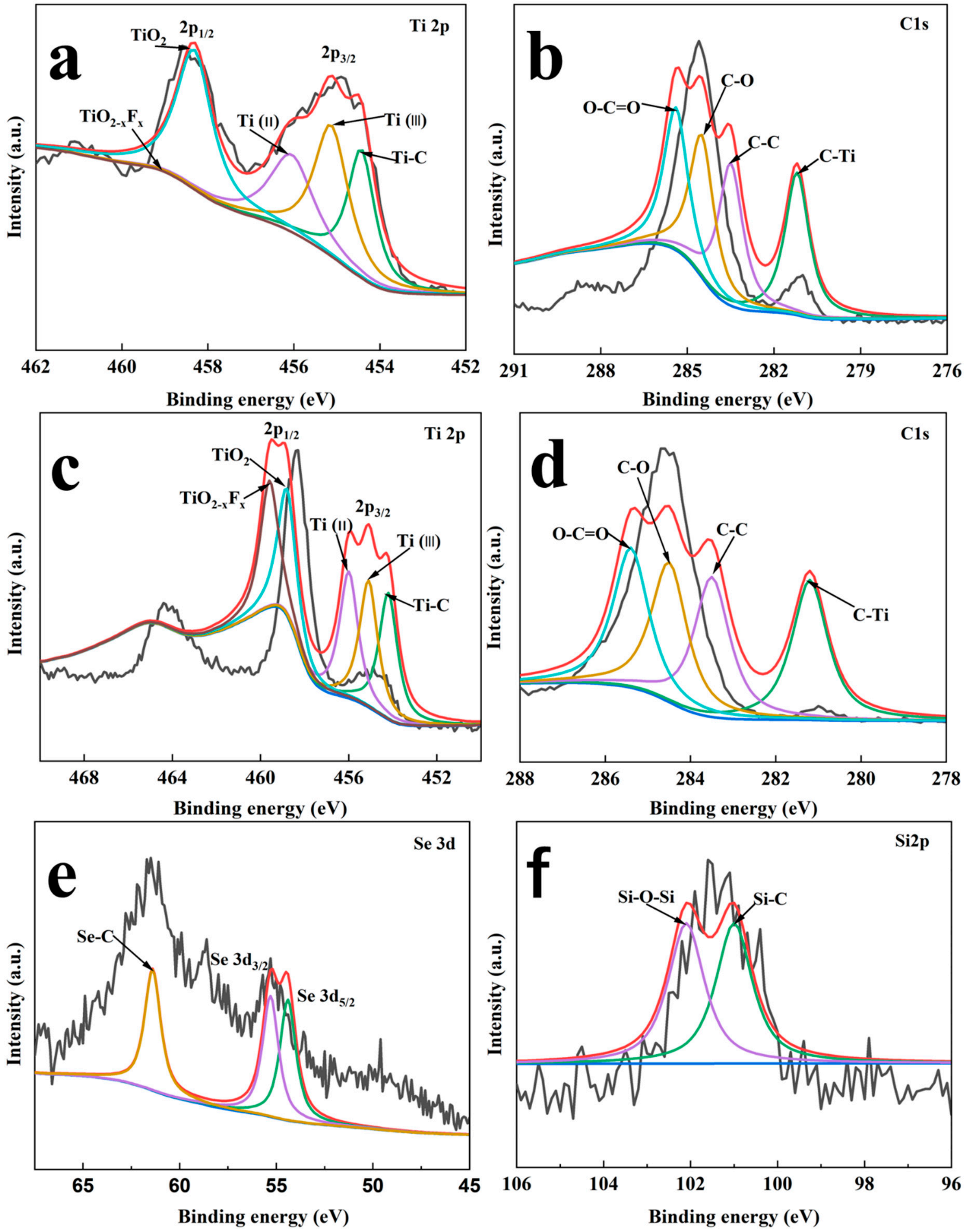

Figure S7 XPS spectrum of MXene-Se and MXene-Se-DOX nanosheets. a, b) Ti 2p and C1s spectrum for MXene-Se; c-f) Ti 2p, C 1s, Se 3d, and Si 2p spectrum for MXene-Se-DOX.

Table S1 In vitro DOX release mechanism study for different formulations based on the Higuchi model.

Sample Code	Release medium	Higuchi [b]	
		K_h	R^2
MXene-Se-DOX	pH 5.5	0.755	0.851
	pH 7.4	0.071	0.661
	pH 7.4 + H_2O_2	1.990	0.916
	pH 7.4 + GSH	2.903	0.901
	pH 5.5 + H_2O_2	6.929	0.919
	pH 5.5 + GSH	8.159	0.895

[b]K_h is the release rate constant, and R^2 is the correlation coefficient.

The general simplified Higuchi model is expressed by the equation given below:

Higuchi: $$M_t = k_h t^{1/2} \quad (1)$$

Where M_t is the release rate at time t and k_h is the rate constant.
